# Serum procalcitonin and CRP levels in non-alcoholic fatty liver disease: a case control study

**DOI:** 10.1186/1471-230X-9-16

**Published:** 2009-02-17

**Authors:** Nevin Oruc, Omer Ozutemiz, Gul Yuce, Ulus S Akarca, Galip Ersoz, Fulya Gunsar, Yucel Batur

**Affiliations:** 1 Gastroenterology Department, Ege University Faculty of Medicine, Izmir, Turkey; 2 Pathology Department, Ege University Faculty of Medicine, Izmir, Turkey

## Abstract

**Background:**

Both C reactive protein (CRP) and procalcitonin (PCT) are well known acute phase reactant proteins. CRP was reported to increase in metabolic syndrome and type-2 diabetes. Similarly altered level of serum PCT was found in chronic liver diseases and cirrhosis. The liver is considered the main source of CRP and a source of PCT, however, the serum PCT and CRP levels in non-alcoholic fatty liver disease (NAFLD) were not compared previously. Therefore we aimed to study the diagnostic and discriminative role of serum PCT and CRP in NAFLD.

**Methods:**

Fifty NAFLD cases and 50 healthy controls were included to the study. Liver function tests were measured, body mass index was calculated, and insulin resistance was determined by using a homeostasis model assessment (HOMA-IR). Ultrasound evaluation was performed for each subject. Serum CRP was measured with nephalometric method. Serum PCT was measured with Kryptor based system.

**Results:**

Serum PCT levels were similar in steatohepatitis (n 20) and simple steatosis (n 27) patients, and were not different than the control group (0.06 ± 0.01, 0.04 ± 0.01 versus 0.06 ± 0.01 ng/ml respectively). Serum CRP levels were significantly higher in simple steatosis, and steatohepatitis groups compared to healthy controls (7.5 ± 1.6 and 5.2 ± 2.5 versus 2.9 ± 0.5 mg/dl respectively p < 0.01). CRP could not differentiate steatohepatitis from simple steatosis. Beside, three patients with focal fatty liver disease had normal serum CRP levels.

**Conclusion:**

Serum PCT was within normal ranges in patients with simple steatosis or steatohepatitis and has no diagnostic value. Serum CRP level was increased in NAFLD compared to controls. CRP can be used as an additional marker for diagnosis of NAFLD but it has no value in discrimination of steatohepatitis from simple steatosis.

## Background

Non alcoholic fatty liver disease (NAFLD) is increasing health problem especially in western countries [1,2]. It is usually associated with co-morbidities including hyperlipidemia, diabetes or metabolic syndrome [3]. Although initially it is considered as benign disorder, now it is accepted that the spectrum of the disease ranges from simple steatosis to steatohepatitis, even to cirrhosis [4]. There are no noninvasive serum markers suggesting or reflecting the disease stage.

Both CRP and PCT are acute phase reactant proteins [5]. They are easy to reach, commonly used, reliable, inexpensive serum markers and extensively used for diagnosis and follow up of several morbidities [5,6]. CRP is synthesized mainly in the liver. The serum CRP level was reported to increase in metabolic syndrome and diabetes [7]. CRP has also been suggested as a predictor of cardiovascular events in patients with metabolic syndrome [7,8]. PCT, a 116-amino acid pro-hormone of calcitonin, is normally synthesized in C cells of thyroid gland. However even thyroidectomised subjects have maintained PCT response during acute inflammation suggesting possible other sources of PCT production including liver and inflammatory cells. PCT was found to be increased in bacterial infections and sepsis [9]. Similarly altered level of serum PCT has been reported in chronic liver diseases and cirrhosis. On the other hand serum PCT levels are not elevated by viral or autoimmune diseases of the liver [10].

The liver is considered the main source of CRP and a source of PCT; however, the serum PCT levels in NAFLD were not investigated previously. Therefore we aimed to study the diagnostic and discriminative role of serum PCT and CRP in NAFLD.

## Methods

Between January 2005 and 2006 all patients admitted to Hepatology outpatient unit with elevated liver function tests, no alcohol history, no drug usage, with negative viral hepatitis and autoimmune serology were further evaluated for NAFLD. Patients with suspected toxic liver disease, cholestatic liver disorders, obstructive jaundice, previously diagnosed Wilson diseases, hemochomatosis, gastrointestinal bypass surgery, systemic disorders and infections were excluded from the study. None of the patients was using statin, corticosteroids or any other medication that known to affect serum CRP levels. Out of total 258 patients evaluated 50 patients were included to the study.

Patients were subjected to general physical examination and routine laboratory investigations. All subjects had normal white blood cell count, urine analysis. Physical examination revealed no sign of infection. Body mass index (BMI) was calculated, ultrasound evaluation was performed for each subject. Liver biopsy was performed when indicated. Histopathological evaluations were performed with an experienced pathologist according to classification suggested by Brunt et al. Briefly, steatosis was classified as S0, no steatosis; S1, < 30% of the hepatocytes in the biopsy with macrovesicular steatosis; S2, 30–60% of the hepatocytes with macrovesicular steatosis; and S3, > 60% of the hepatocytes with macrovesicular steatosis. Nonalcoholic steatohepatitis was diagnosed when the presence of steatosis with hepatocellular ballooning and/or necrosis was associated with neutrophil polymorphonuclear cell infiltration. A total of 50 consecutive patients (33 males and 17 females, age 54.7 ± 14.8 years) with liver biopsy proven NAFLD were included to the study. Results were compared with healthy volunteers (n 50, mean age 45.1 ± 4.3). Ultasonoghraphic evaluation of control subjects confirmed that there was no NAFLD. Liver function tests were normal in controls.

Blood samples were withdrawn at the same day with definitive diagnosis. Fasting blood samples were obtained and used for determination of glycemia, insulinemia, cholesterol, HDL- and LDL-cholesterol, and liver function tests. Insulin resistance was determined by using a homeostasis model assessment of insulin resistance (HOMA-IR) [11]. Serum CRP was measured with nephalometric method. PCT was measured in plasma samples with Kryptor based system as suggested by the company. (B.R.A.H.M.S.-Diagnostica, Berlin, Germany). Lowest detection limit was 0.01 ng/ml. Values of PCT levels > 0.5 ng/ml were considered as abnormal, as proposed by the manufacturer.

Study was performed with the approval of Ege University local ethics committee and carried out in compliance with the Helsinki Declaration. Written informed consent was given by all subjects. Results were given as mean ± SEM. SPSS statistical package (SPSS software, PC version 11.5; SPSS, Chicago IL) was used for statistical analyses. Statistical analysis was based on paired Student's t-test and the nonparametric Kruskall-Wallis test. ROC analysis was conducted to calculate sensitivity and specificity of the markers. P < 0.05 was considered as significant.

## Results

A total of 50 consecutive patients with liver biopsy proven NAFLD were included to the study. Results were compared with healthy volunteers (n:50, mean age 45.1 ± 4.3). Age and gender distribution was similar between NAFLD group and healthy controls. Patient's and control's characteristics were given in Table 1.

**Table 1:** General characteristic of NAFLD patients.

	Steatohepatitis (n 20)	Diffuse Steatosis(n 27)	Focal Fatty Liver (n 3)	Controls (n 50)
Age(years)	54.1 ± 13.2	45.1 ± 6.3	56.1 ± 3.1	43.8 ± 17.5
BMI (kg\m2)	30.6 ± 1.4	34.7 ± 4.5	26.7 ± 0.5	28.1 ± 5.3*
ALT (U\l)	68.0 ± 12.7	69.6 ± 16.2	42.0 ± 15.0	28.4 ± 1.1*
AST (U\l)	54.1 ± 13.2	45.2 ± 6.3	68.0 ± 7.0	27.3 ± 1.2*
GGT	62.8 ± 9.9	53.2 ± 5.9	48.0 ± 8.1	49.3 ± 8.5
Cholesterol (mg\dl)	203.8 ± 15.2	174.0 ± 13.3	167.0 ± 10.2	171 ± 10.1
Triglyceride (mg\dl)	214.0 ± 47.4	134.8 ± 15.9	73.0 ± 15.2	90 ± 10.5
HOMA-IR	6.2 ± 1.4	4.9 ± 0.9	1.5 ± 0.8	1.4 ± 0.8*
USG Steatosis Grade	2.04 ± 0.01	1.87 ± 0.22	0	0

*BMI of healthy controls was lower than steatohepatitis and steatosis patients. Liver function tests, HOMA-IR value were significantly higher in NAFLD groups compared to controls (p < 0.01)

**Figure 1:**
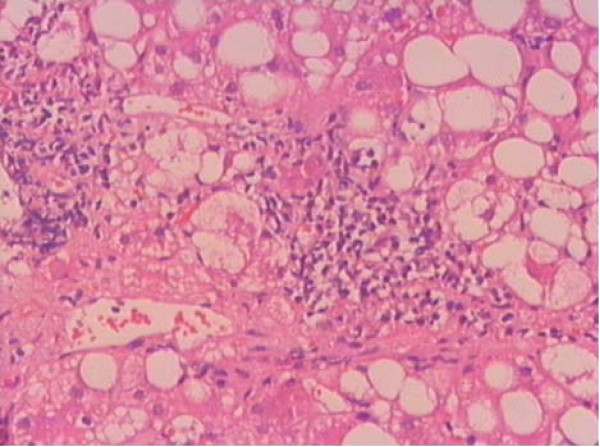
**Portal inflammation is a diagnostic finding in NASH**. Histopathologic section was obtained from one of patients with steatohepatitis. [HE stain, ×40]

Liver biopsy proved the diagnosis of steatohepatitis in 20, diffuse steatosis in 27 and focal fatty infiltration in 3 subjects (Figure 1). Patients with steatohepatitis or steatosis had high HOMA-IR values demonstrating the presence of insulin resistance. Serum AST and ALT concentrations, BMI, and HOMA-IR values were significantly higher in NAFLD group compared to controls (p < 0.01) (Table 1).

Serum PCT levels were within normal ranges in both NAFLD and control group. There was no significant difference between the steatohepatitis, steatosis and control group in PCT levels (0.06 ± 0.01 and 0.04 ± 0.01 versus 0.06 ± 0.01 ng/ml respectively). Serum CRP levels were significantly increased in patients with steatohepatitis and steatosis compared to controls. (5.2 ± 2,5 and 7.5 ± 1.6 versus 2.9 ± 0.5 mg/dl respectively p < 0.01). However there was no significant difference between steatohepatitis and steatosis patients in serum CRP levels. Three patients with focal fatty liver disease had normal serum CRP and PCT levels (Table 2)

**Table 2:** Serum PCT and CRP levels in study groups.

	CONTROLS		NAFLD		P value
	(n 50)	Focal Fatty liver (n3)	Steatohepatitis (n20)	Steatosis (n27)	

PCT	0.06 ± 0.01	0.03 ± 0.01	0.06 ± 0.01	0.04 ± 0.01	ns
CRP	2.90 ± 0.50	1.70 ± 0.70	5.20 ± 2.50*	7.50 ± 1.60*	< 0.01

Serum CRP levels were significantly higher in simple steatosis and steatohepatitis groups compared to healthy controls (*p < 0.01). There were no significant differences between groups in serum PCT levels.

When patients were divided into two groups according to presence of insulin resistance, insulin resistance did not affect the CRP and PCT levels in NAFLD patients (Table 3). PCT was not correlated with liver function tests, BMI or presence of insulin resistance. ROC curve analysis reveled that serum CRP levels above 3 has 73% sensitivity and 68% specificity for NAFLD.

**Table 3:** Effects of insulin resistance on serum PCT and CRP levels.

	STEATOSIS			STEATOHEPATITIS		
	Insulin Resistance -	Insulin Resistance +	P value	Insulin Resistance -	Insulin Resistance +	P value

PCT	0.05 ± 0.01	0.06 ± 0.02	0.71	0.06 ± 0.01	0.04 ± 0.02	0.12
CRP	6.90 ± 2.50	5.60 ± 1.10	0.59	5.90 ± 1.20	5.20 ± 1.10	0.67

NAFLD patients grouped according to presence of insulin resistance. HOMA-IR > 2.5 was considered as insulin resistance. Insulin resistance does not determine the serum PCT or CRP levels.

## Discussion

Diagnostic and prognostic value of serum PCT level in liver disorders has been evaluated in some studies with controversial results [12]. In patients with decompensated liver cirrhosis high PCT level was sensitive and specific tool for the initial diagnosis of bacterial infection [13]. On the other hand acute alcoholic hepatitis as well as acute viral hepatitis on cirrhotic background without proved bacterial infection induce mild elevation of serum PCT levels [14,15]. Our study revealed that serum PCT level was within normal ranges in both steatohepatitis and steatosis patients for the first time in the literature. This finding suggests that PCT is a specific marker for infection and does not necessarily increase in fatty infiltration or inflammation located in the liver. Although we did not evaluate the expression pattern of PCT we might speculate that liver is not the main source of PCT production. PCT was not correlated with liver function tests, BMI or presence of insulin resistance either.

We also evaluated the clinical usefulness of CRP in the diagnosis of NAFLD. There were limited number of studies implying that serum CRP was elevated in NAFLD [16-18]. CRP has short life around 18 hours and the elevation of serum CRP usually reflects its synthesis in response to a pathological process [19]. CRP is therefore considered as a useful nonspecific biochemical marker of chronic inflammation [19]. Our study confirmed that the increase in circulating CRP levels could be by itself a marker of the presence of NAFLD. The C reactive protein response however alone has no diagnostic specificity. Due to its low specificity, C reactive protein values can really only be interpreted when all other clinical and laboratory information is available. We therefore suggest that serial measurements of CRP can be helpful in clinical management and follow up of NAFLD patients.

In clinical trials CRP elevation was related to metabolic syndrome and its components. Although the liver is main source of the CRP production, fat tissue, especially visceral fat significantly contributes to CRP production [20]. But there is no evidence about the synthesis of PCT by adipose tissue. It is also well known that CRP concentration was elevated in severely obese patients but this elevation was moderate and not related to metabolic syndrome, diabetes, and more importantly to steatohepatitis [20]. In concordance with that our study failed to show any association between the presence of insulin resistance, and serum CRP or PCT levels. Since both steatohepatitis and steatosis group was obese in our study, similarly elevated CRP probably was related to adiposity.

Another proposed mechanism for CRP response in NAFLD is the elevation in acute phase cytokines. IL-6 for example, is a potent stimulator for CRP synthesis. Haukeland et al. reported that both patients with simple steatosis and steatohepatitis had significantly raised serum levels of IL-6 compared with healthy controls [16]. Although we did not measure the serum level of inflammatory cytokines, this might explain the equally elevated serum CRP levels in both steatohepatitis and steatosis groups in our study. Unfortunately, this common pathogenesis decreases the discriminative role of CRP for steatohepatitis. Acute effects of those cytokines on PCT levels were already demonstrated in experimental studies, but this affect is obviously less then expected in NAFLD [21]

## Conclusion

Serum PCT was within normal ranges in patients with simple steatosis or steatohepatitis and has no diagnostic value in NAFLD. CRP can be used as an additional marker for diagnosis of NAFLD but it has no value in discrimination of steatohepatitis from simple steatosis. Obesity but not the insulin resistance is determining factor for the serum CRP levels. Serial measurements of CRP levels in obese patients might increase its diagnostic value in NAFLD and should be evaluated in further studies.

## Abbreviations

NAFLD: Non alcoholic fatty liver disease; CRP: C reactive protein; PCT: Procalcitonin; HOMA-IR: Homeostasis model assessment of insulin resistance. BMI: Body mass index

## Competing interests

The authors declare that they have no competing interests.

## Authors' contributions

NO planned and coordinated the study, NO and OO prepared manuscript; GY evaluated the pathological sections for diagnosis. USA, GE, FG, YB were involved in the diagnosis and recruitment of cases. All authors read and approved the final manuscript.

## Pre-publication history

The pre-publication history for this paper can be accessed here:

http://www.biomedcentral.com/1471-230X/9/16/prepub
